# Association between Odontogenic and Maxillary Sinus Conditions: A Retrospective Cone-Beam Computed Tomographic Study

**DOI:** 10.3390/jcm10132849

**Published:** 2021-06-27

**Authors:** Piotr Kuligowski, Aleksandra Jaroń, Olga Preuss, Ewa Gabrysz-Trybek, Joanna Bladowska, Grzegorz Trybek

**Affiliations:** 1 Department of Oral Surgery, Pomeranian Medical University in Szczecin, Al. Powstańców Wlkp. 72, 70-111 Szczecin, Poland; piotr.kuligowski@pum.edu.pl (P.K.); jaronola@gmail.com (A.J.); olga.preuss@pum.edu.pl (O.P.); 2 Department of Diagnostic Imaging and Interventional Radiology, Pomeranian Medical University, Unii Lubelskiej 1 St., 71-242 Szczecin, Poland; ewa_gabrysz@wp.pl; 3 Department of General and Interventional Radiology and Neuroradiology, Wroclaw Medical University, Borowska 213 St., 50-556 Wroclaw, Poland; joanna.bladowska@umed.wroc.pl

**Keywords:** odontogenic infections, cone-beam computed tomography, maxillary sinus, sinusitis

## Abstract

Odontogenic infections can directly trigger maxillary sinusitis. CBCT is an excellent choice for precise examination of maxillary sinuses and hard tissues within the oral cavity. The objective of this retrospective and the cross-sectional study was to analyze the influence of odontogenic conditions on the presence and intensity of maxillary sinus mucous membrane thickening using CBCT imaging. Moreover, periodontal bone loss and anatomic relationship between adjacent teeth and maxillary sinuses were assessed to evaluate its possible impact on creating maxillary thickening. The study sample consisted of 200 maxillary sinuses of 100 patients visible on CBCT examination with a field of view of 13 × 15 cm. The presented study revealed a significant influence of periapical lesions, inappropriate endodontic treatment, severe caries, and extracted teeth on the presence of increased thickening of maxillary sinus mucous membrane. In addition, an increase in the distance between root apices and maxillary sinus floor triggered a significant reduction of maxillary sinus mucous membrane thickening. The presence of periodontal bone loss significantly increases maxillary sinus mucous membrane thickening.

## 1. Introduction

The maxillary sinus is a pyramid-shaped cavity located in the maxilla with a mean volume of 12.5 mL [1,2]. The maxillary sinus is connected with the nasal cavity by the ostium. Its main functions include e.g., reducing the overall weight of the skull, and contributing to the olfactory process [3]. The maxillary sinus is lined with a thin respiratory mucous membrane that firmly adheres to the periosteum, also known as the Schneiderian membrane [4]. Healthy maxillary sinus mucosa may not be visible on radiographs, and its thickness does not exceed 2 mm [5]. Mucosal thickening greater than 2 mm is considered pathological [6]. 

Moreover, it is the most common signs of maxillary sinusitis visible on X-ray [6]. Mucosal thickening is a general defensive reaction of the maxillary sinus to the inflammatory process, which results in hypertrophy of the epithelial cells [7]. This process can be triggered by odontogenic infection, paranasal sinusitis, chemicals, allergy, and bronchial asthma [8]. In dentistry, maxillary sinus imaging is most commonly used in panoramic radiography, ensuring visualization of several different anatomical structures and relatively low radiation dose absorbed during examination [9]. However, the complexity of the oral and maxillofacial region may cause the superimposition of neighboring structures. Moreover, vertical and horizontal magnification, lack of cross-sectional view are other disadvantages of panoramic radiography [10]. The conventional radiological examination provides insufficient reliability. Therefore, a much more precise diagnostic method is needed to evaluate the maxillary sinus and maxillofacial region properly. CBCT (cone beam computed tomography) is considered the definite and proper tool for imaging structures within the maxillofacial region, including maxillary sinus [11]. CBCT delivers exceptional diagnostic accuracy in examining teeth, alveolar bone, and maxillary sinus morphology [12]. In addition, CBCT provides reduced radiation dose and lower cost of examination than traditional CT (computed tomography) [13].

Thickening of the maxillary sinus can also be caused by the occurrence of retention cysts (RC) or pseudo-cysts of the maxillary sinus. These are pathologic conditions whose etiology is not necessarily related to odontogenic infections, and they manifest as thickening of the maxillary sinus mucosa [14]. These conditions are asymptomatic in most cases; however, when they block the sinus ostium or lead to sinusitis, they can become a problem for the patient [15,16,17,18].

Odontogenic diseases can cause maxillary sinus infections. The incidence of odontogenic sinusitis is estimated at approximately 10–12% of all maxillary sinus infections [19]. However, according to the most recent studies using CBCT and CT, the prevalence of odontogenic sinusitis may reach 40% [20]. Odontogenic sinusitis may be localized and manifest as a mucosal thickening in the maxillary sinus restricted only to the vicinity of the infected tooth [21]. Previous studies suggest that apical periodontitis, periodontal disease, trauma, surgical procedures concluded in maxilla such as extraction of the teeth, endodontic treatment, retained teeth can cause the maxillary sinus infection [11,22,23]. Some researchers indicate that proximity of root apices to the maxillary sinus may increase the potential impact on the formation of odontogenic sinusitis [24]. In addition, the maxillary sinus floor may expand deeply into the alveolar process of the maxilla, creating maxillary recess, which is present in approximately 50% of the population [2,9]. The maxilla mainly consists of spongy bone. Therefore, pathological bacteria and toxins from the oral cavity may directly infiltrate the maxillary sinus. Most cases of odontogenic sinusitis include unilateral infection. However, bilateral cases also occur [25]. Bacterial flora in odontogenic sinusitis contains mainly anaerobic microorganisms from Peptosteptococcus, Prevotella, and Porphryromonas species [26].

Moreover, this type of infection lacks characteristic bacteria responsible for paranasal sinusitis, such as Haemophilus influenza and Moraxella catharrhalis [21]. Odontogenic sinusitis may be resistant to conventional paranasal sinusitis therapy. Therefore, the correct identification of underlying dental conditions is highly recommended [27].

The objective of this retrospective and the cross-sectional study was to analyze the influence of odontogenic conditions on the presence and intensity of maxillary sinus mucous membrane thickening using CBCT imaging. Moreover, periodontal bone loss and anatomic relationship between adjacent teeth and maxillary sinuses were assessed to evaluate its possible impact on creating maxillary thickening. 

## 2. Materials and Methods

This retrospective radiological study was conducted at the Department of Oral Surgery of the Medical University following the decision of the Bioethical Committee No. KB-0012/271/09/18. One hundred consecutively enrolled patients meeting inclusion and exclusion criteria were included in the study. Patients underwent CBCT imaging for the following purposes: assessment of impacted teeth, planning of dental implant treatment, pre-prosthetic evaluation, endodontic diagnostic, temporomandibular joints disorders. To the presented study were qualified one hundred adult patients with visible two hundred maxillary sinuses. The exclusion criteria were as follows: allergies, common cold or infections of upper respiratory tracks in the last four weeks, diagnosed acute or chronic maxillary sinusitis, asthma, osteoporosis, traumas, and neoplastic diseases in the area of the cranial facial portion due to previous diagnosis mentioned in dental chart. 

CBCT examinations were acquired using CRANEX^®^ 3Dx (Soredex, Tuusula, Finland) and then analyzed retrospectively. The images were taken using standard parameters (89 kVp, 7–8 mA, pixel size 0.085 mm, the field of view 15 cm × 13 cm). Only high-quality images providing visibility of both maxillary sinuses with a horizontal plane parallel to the floor, without any distortion, superimposition and artifacts were included in the study. CBCT examination analysis was conducted using software OnDemand3DTM Dental on the monitor with resolution 1920 × 1200 pixels in a room with the lights dimmed. All CBCT images were evaluated twice with one-month intervals by the same observer.

### 2.1. Assessment of Odontogenic Condition

**Figure 1:**
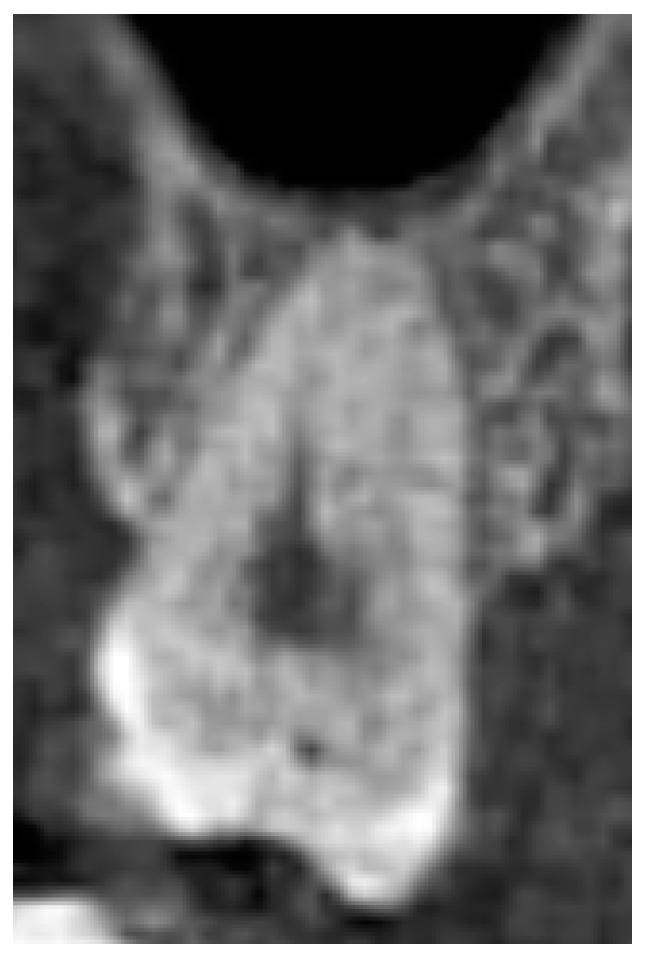
H—the healthy teeth.

**Figure 2:**
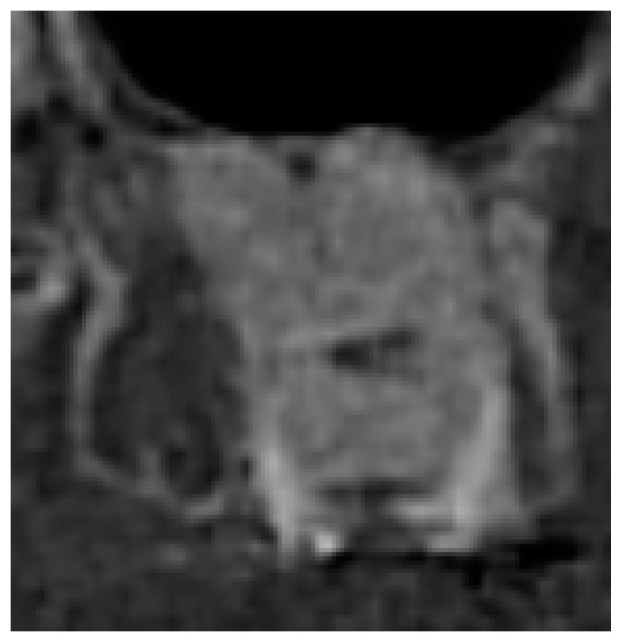
I—the impacted teeth.

**Figure 3:**
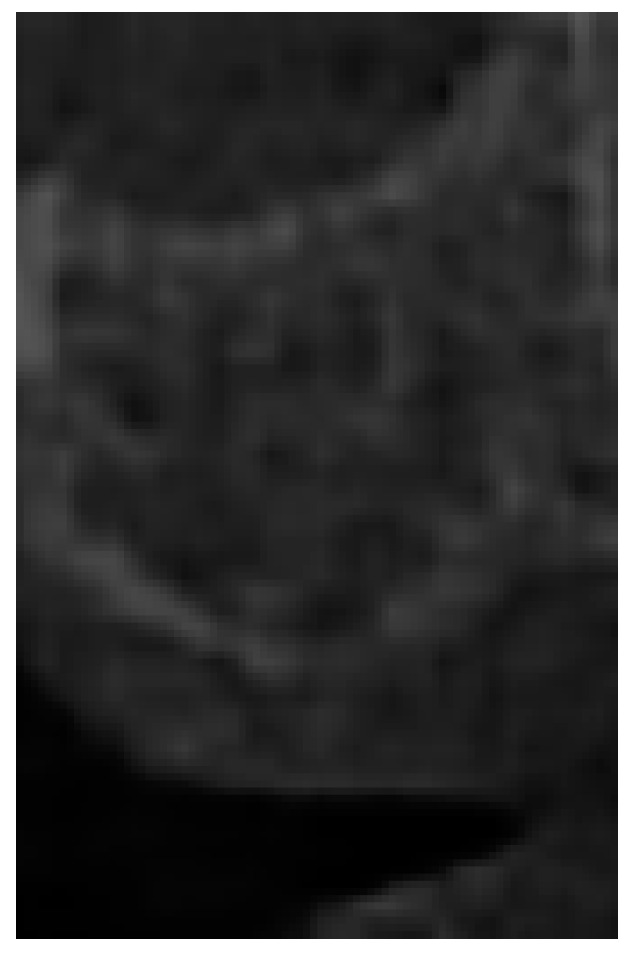
R—the removed teeth.

**Figure 4:**
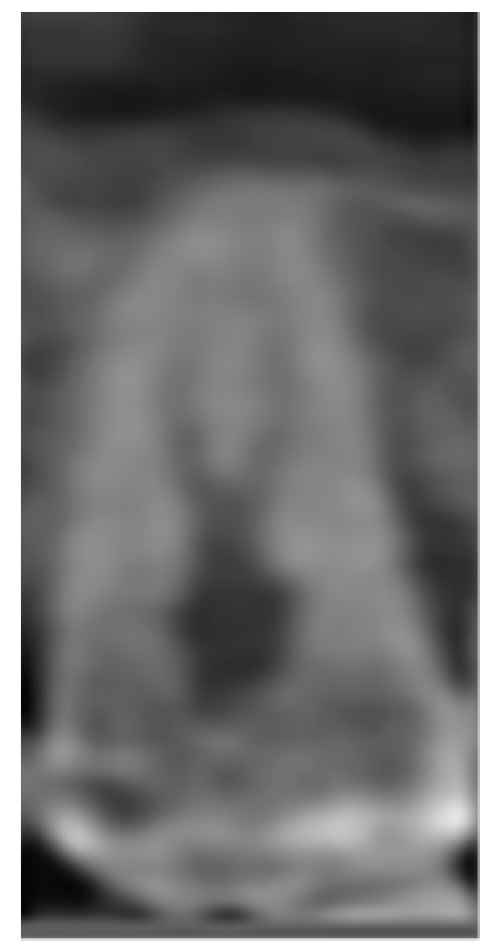
C—the teeth with severe caries infection.

**Figure 5:**
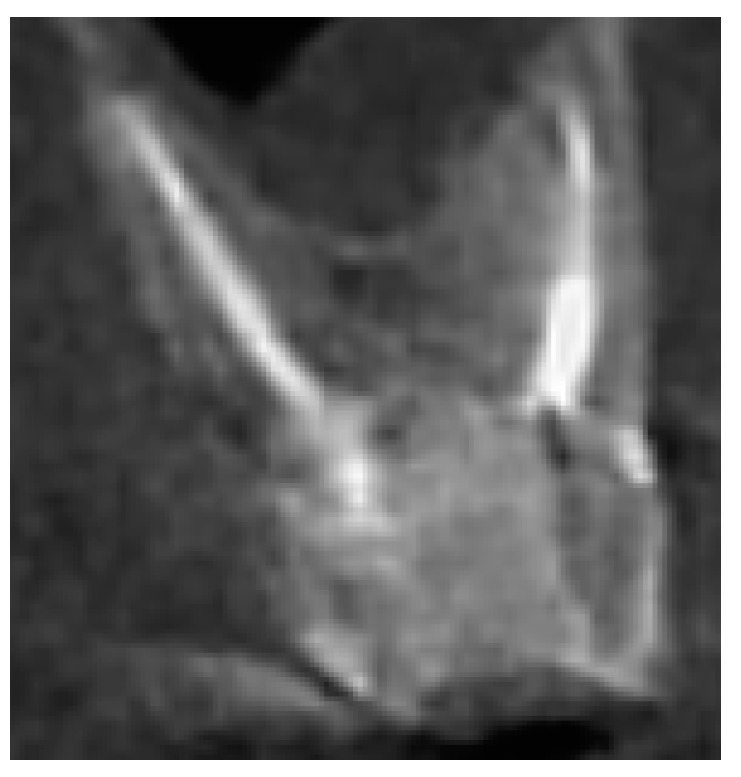
E—the teeth subjected to successful endodontic treatment.

**Figure 6:**
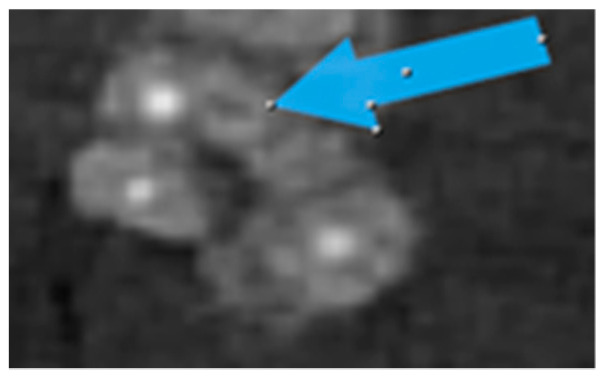
NE—the teeth subjected to unsuccessful endodontic treatment.

**Figure 7:**
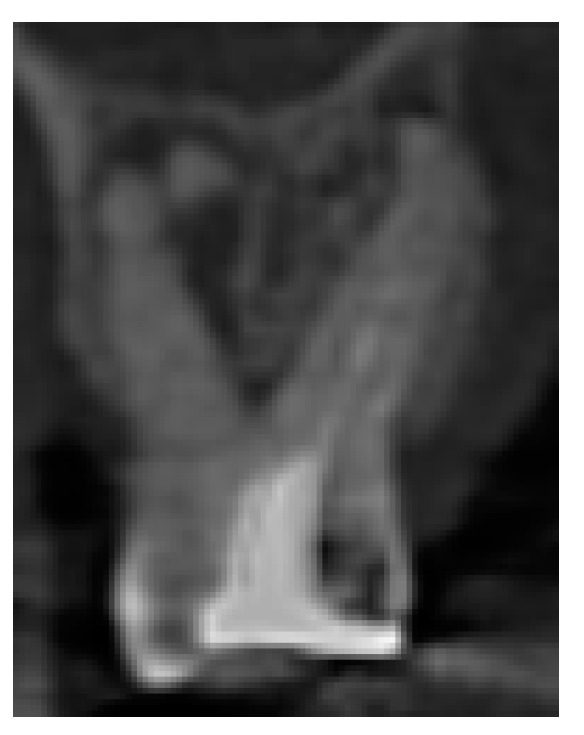
P—the teeth with periapical lesion.

Each tooth was evaluated in the axial, coronal, sagittal, and cross-sectional view of CBCT. Then every single tooth was classified into one of the following group: H—healthy teeth (Figure 1), I—impacted teeth (Figure 2), R—removed teeth (Figure 3), C—teeth with severe caries infection that proceed more than half of dentin (Figure 4), E—teeth subjected to successful endodontic treatment (Figure 5), NE—teeth subjected to unsuccessful endodontic treatment (Figure 6). The tooth was classified to this group if there was one of the following: inadequate filling of the canal, improper coronal seal, instrumentation complications (ledges, perforations, or separated instruments), untreated canals, overextension of root canal material [28]. P—teeth with the periapical lesion (Figure 7).

### 2.2. Assessment of Anatomic Relation between Teeth and Maxillary Sinus

**Figure 8:**
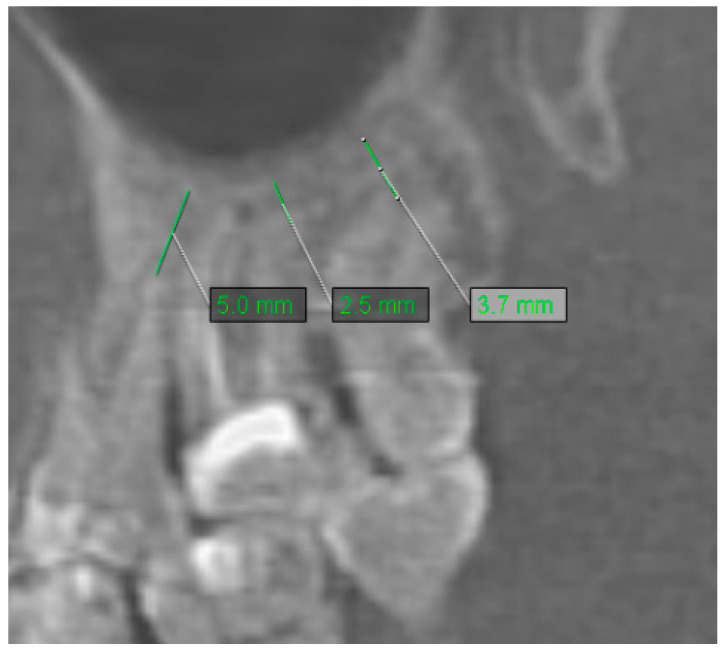
Sample measurement of distance of the teeth to maxillary sinus.

The distance between root apices of maxillary teeth was evaluated in the axial, coronal, sagittal, and cross-sectional views of CBCT. From this analysis, were excluded removed teeth–R. Only the lowest distance to maxillary sinus was recorded for each examined tooth. If the tooth was in contact with the maxillary sinus, the recorded distance equaled 0. Sample measurement of the distance of the teeth to the maxillary sinus is shown in Figure 8.

### 2.3. Assessment of Periodontal Bone Loss

**Figure 9:**
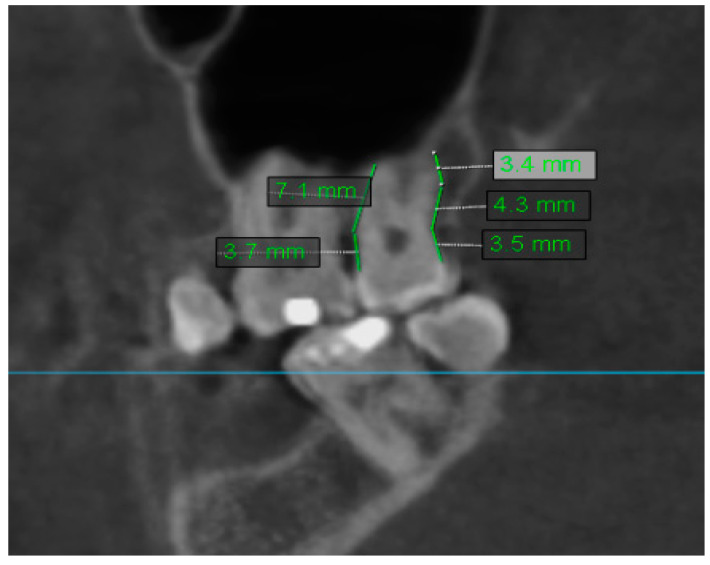
Sample measurement of periodontal bone loss.

Periodontal bone loss was evaluated on each tooth’s mesial and distal sides in the sagittal or coronal view of CBCT. From this part of the analysis were excluded R—removed teeth and I—impacted teeth. The alveolar crest ridge is considered the referential point to which the final periodontal ligaments are attached [29]. The physiological distance between cementoenamel junction (CEJ) and alveolar crest ridge equals 1 mm. First, the distance between CEJ and alveolar crest ridge was measured to evaluate periodontal bone loss. Subsequently, 1 mm was subtracted from this value. Then the obtained difference was divided by total root length, which was measured as the distance from CEJ to the root apex. The result was featured as a percentage value. Figure 9 presents an example of measuring periodontal bone loss.

### 2.4. Assessment of Mucosal Thickening

**Figure 10:**
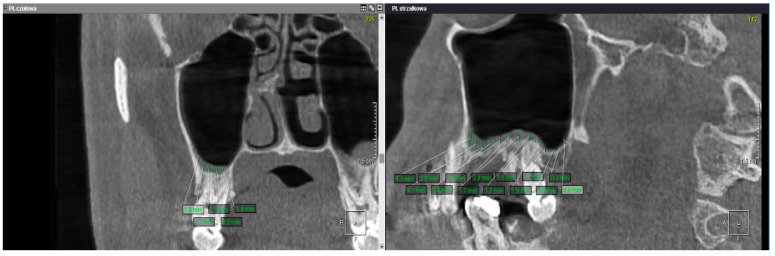
Sample measurement of mucosal thickening.

Thickening of the maxillary sinus mucosa was assessed separately for each tooth in the sagittal, coronal, and cross-sectional view of CBCT. In each maxillary sinus, mucosal thickening was analyzed at the highest thickness from the maxillary sinus floor in the projection of examined tooth. Thus, in every maxillary sinus, there were six measurement points. In this part of the study, every group of teeth was examined: H, I, R, C, E, NE, P. If the patient had several missing teeth and the previous position of the missing teeth seemed ambiguous, it was assumed that in the edentulous sites distances between premolars roots were set at 7 mm and distances between molars roots was set at 8 mm [8]. Sample measurement of mucosal thickening is shown in Figure 10. Results of analysis of mucosal thickening were also classified as [29]:

Grade 1—0–2 mm–normal sinus mucosa;

Grade 2—2–10 mm–moderate mucosal thickening;

Grade 3 > 10 mm–severe mucosal thickening.

### 2.5. Methodology of Statistical Analysis

Statistical analysis was conducted using the R program, version 3.5.2 (R Core Team, Vienna, Austria, 2018). Standard measures of location were used to describe quantitative variables: quartiles, arithmetic mean, median, and measures of variability: standard deviation, minimum, and maximum. Qualitative variables were defined by: number and percentage of occurrences of each value. 

The comparative analysis of qualitative variables was performed using Fisher exact test, where low expected values appeared.

In the non-normality of distribution in two groups, quantitative variables were carried out using the Mann–Whitney test. Kruskal–Wallis test was used in the absence of normality of distribution in the groups. When statistically significant differences were detected in groups with non-normality of distribution, Dunn’s test was used. Correlations of quantitative variables were analyzed in the absence of normality of distribution of the variables using the Spearman correlation coefficient. Adjusted effect analysis on quantitative variables was performed using a linear regression method. A significance level of *p* = 0.05 was assumed in the study. All *p* values below 0.05 were interpreted as statistically significant.

## 3. Results

The study enrolled 100 patients who underwent CBCT covering 200 maxillary sinuses. The study group included 50 men and 50 women. Researched cohort age ranged from 22 to 84 years; the mean age was 46.63 ± 15.5. The characteristics of the study group are listed in Table 1.

### 3.1. Comparison Analysis of Mucosal Thickening According to Odontogenic Condition

This part of the study was conducted using the Kruskal–Wallis test due to non-normal data distribution; posthoc analysis was performed with Dunn’s test. Statistical analysis shows a significant relationship between the odontogenic condition of teeth and the presence of maxillary sinus mucosa membrane thickening in the projection of examined tooth (*p* < 0.001). The average thickening of the maxillary sinus mucosa was, respectively, in Group P (teeth with periapical lesions)–12.35 ± 10.12 mm, in Group NE (teeth subjected to unsuccessful endodontic treatment)–6.86 ± 8.9 mm, in Group R (removed teeth)–6.46 ± 9.07 mm, in Group C (teeth with severe caries infection)–6.38 ± 7.07 mm, in Group I (impacted teeth)–3.8 ± 4.99, in Group E (teeth subjected to successful endodontic treatment)–3.25 ± 4.72 mm and in Group H (healthy teeth)–2.87 ± 5.04 mm. The highest mean values of mucosal thickening were confirmed in teeth with periapical lesions and the lowest in healthy teeth. Significantly greater thickening of the maxillary sinus mucosa occurred in Group P compared to Groups NE, R, C, I, E, H. In addition, significantly higher values of thickening of the maxillary sinus mucosa were found in the NE Group concerning the R, C, I, E, H groups. More significant thickening of the maxillary sinus mucosa occurred in Group R compared to Groups C, I, E, H. Significantly greater thickening of the maxillary sinus mucosa was found in Group C concerning Groups I, E, H. The exact information on this issue is presented in Table 2.

### 3.2. Comparison Analysis of Mucosal Thickening According to Periodontal Bone Loss

This part of the study was performed using Spearman’s rank correlation coefficient due to non-normal data distribution. Statistical analysis revealed a positive correlation between mean alveolar bone loss and the maxillary sinus mucosa thickening. Statistically significant correlation was obtained with Teeth 17 (*p* < 0.001), 14 (*p* = 0.017), 13 (*p* = 0.02), 24 (*p* = 0.003), and 27 (*p* = 0.045). Teeth 17 and 24 showed weak correlation strength, while Teeth 14, 13, 27 showed very weak correlation. The greater the loss of alveolar bone, the greater the thickening of the maxillary sinus mucosa. The exact data of analysis is presented in Table 3.

### 3.3. Comparison Analysis of Mucosal Thickening According to Anatomic Relation between Teeth and Maxillary Sinus

The presented analysis demonstrated a negative correlation between the average distance of the tooth root apex to the floor of the maxillary sinus and the average thickening of the maxillary sinus mucosa. Statistically significant correlation was obtained with Teeth 16 (*p* = 0.009), 15 (*p* = 0.014), 27 (*p* = 0.004). Teeth 16 and 27 showed weak correlation strength, while Tooth 15 showed a very weak correlation. The greater the distance between the root apex of the tooth and the bottom of the maxillary sinus, the smaller the thickening of the mucous membrane of the maxillary sinus. The exact data of analysis is presented in Table 4.

### 3.4. Comparative Analysis of the Average Mucosal Thickening, Classified in a Three-Grade Scale and Odontogenic Condition

In this part of the study, the average mucosal thickening was classified and analyzed on a three-point scale. Grade 1 included mean thickening of the maxillary sinus mucosa less than 2 mm. The average thickening of the mucosa in Grade 2 ranged from 2 mm to 10 mm. Whereas in Grade 3, the average mucosal thickening exceeded 10 mm. The general prevalence of mucosal thickening was in Grade 1—48%, in Grade 2—36%, and Grade 3—16%. Individual grades of mucosal thickening differed significantly in the distribution of the teeth groups (*p* < 0.001). The highest percentage of teeth with periapical lesions were distributed in Grade 3 of mucosal thickening. Grade 2 mainly consisted of the teeth affected by severe caries, while Grade 1 had an enormous number of healthy teeth. The exact data of analysis is presented in Table 5.

## 4. Discussion

For more than a century, maxillary bone infections have been a known cause of maxillary sinusitis, but this fact is often overlooked, and the diagnostic process is closely focused on the natural ostium of these structures [30]. Moreover, it is advisable to perform oral cavity sanitation before surgical intervention [31]. However, despite the widely recognized impact of odontogenic infections on the formation of maxillary sinus inflammation, there is a lack of agreement among authors as to the primary cause of odontogenic sinusitis [32].

There is a great deal of controversy about the clinical symptoms that can occur in thickening the maxillary sinus mucosa. In the Som study, the mucous membrane of the maxillary sinus should not be visible in the radiological examination, and its thickening is considered a pathological condition [33]. However, according to the team of Rak et al., thickening of the maxillary sinus mucosa below 3 mm most often remains asymptomatic [34]. According to studies by Phothikhun et al., in most cases of thickening of the maxillary sinus mucosa within 5 mm, the patient did not experience any clinical symptoms [11]. The team of researchers Savolainen et al. showed that the occurrence of a slight thickening of the maxillary sinus mucosa not exceeding 2 mm is a frequent radiological image in asymptomatic patients [35].

Moreover, the definition of the radiological image of the odontogenic maxillary sinus is not entirely established [5]. There is no complete agreement as to the value of mucosal thickening considered as pathological. The researchers Phothikhun et al. assumed that pathological thickening of the maxillary sinus mucosa occurs when its thickness exceeds 1 mm [11]. Most researchers, including this study, consider the thickness of the maxillary sinus mucosa exceeding 2 mm to be a pathological value [4,12,29].

In the present study, maxillary sinus mucosal thickening was present in 52%, which agrees with the investigators’ results who also used CBCT diagnosis in the study. The more significant thickening of the maxillary sinus mucosa was present; the more sensitive and accurate radiological diagnosis was used. Furthermore, according to Vallo et al., 30% more potential periapical lesions are visible in CBCT imaging [23]. The detection rate of maxillary sinus mucosal thickening is four times higher than in conventional two-dimensional radiographs [23].

Most researchers found a significant relationship between the presence of periapical lesions in teeth in the maxilla and the thickening of the maxillary sinus mucosa, which implies with the current study [4,29,36]. After tooth pulp necrosis, intense bacterial infectious agents, such as collagenase, lysosomal enzymes, and toxins promote bacteria’s spread to periapical tissues [37]. The mechanism of inflammatory changes in the maxillary sinus caused by the proximity of periapical lesions is explained by the spread of bacteria, toxins, and proinflammatory cytokines through the thin porous maxillary bone.

The classification used in the present study was as follows: healthy teeth, extracted teeth, impacted teeth, teeth with deep carious lesions, properly endodontically treated teeth, improperly endodontically treated teeth, dental implants, and teeth with periapical lesions. The classification used is more extensive and comprehensive, which affects the accuracy of the results. Discrepancies in the study results may be due to the use of different imaging methods, the age difference of the study group, and the personal characteristics of the study group. A team of authors Shanbhag et al. found that periapical lesions are most common in molars [36]. Similar results were obtained in the present study, where the prevalence of periapical lesions was highest at Teeth 26, 17, and 27. The prevalence and extent of sinus mucosal thickening depend on the size of periapical lesions [38].

According to Lu et al., the thickening of the maxillary sinus mucosa increases significantly with the increased number of teeth with periapical lesions, confirming the present study results [38]. According to Sheikhi et al. and Goller-Bulut et al., mucosal thickening could be significantly affected by the presence of teeth subjected to unsuccessful endodontic treatment, as well as the teeth with severe caries infection [4,39]. The formation of mucosal thickening in the area of teeth incorrectly endodontically treated is explained by the fact that during root canal treatment, endodontic tools can perforate deep into the maxillary sinus, which may subsequently push the endodontic rinses, sealants, or canal filling materials inside the maxillary sinus [40].

In this study, the teeth most commonly affected by caries and subjected to inadequate endodontic treatment were the upper first molars. The team of Aksoy and Orhan came to similar conclusions [29]. They found that upper first molars were most often subjected to pathological processes. The authors explained this because these teeth are more susceptible to the carious process, pulp disease, and consequently endodontic treatment because they are the first intruding lateral teeth. Furthermore, endodontic treatment of this tooth is complicated due to the complex anatomical structure of the canals, which consequently increases the percentage of improperly endodontically treated first upper molars [41].

There are also reports in the literature of the possibility of allergic reactions to the most commonly used rinses in endodontics [40]. A team of researchers Aksoy and Orhan proved that the prevalence and extent of the maxillary sinus mucosa thickening increase with the number of teeth removed, compliant with the current study [29]. Presumably, most teeth qualified for extraction are a potential source of odontogenic infection that could cause inflammation inside the maxillary sinus before surgery. According to the observations of Block and Dastoura, the removal of highly damaged teeth, which causes mucosal thickening, reduces the severity of the thickening, although it does not eliminate the process, which is in agreement with the presented study [42]. No significant impact of teeth treated endodontically on the increase of mucosal thickening was found in the current study. In addition, these conclusions are consistent with the results of the team of Aksoy and Orhan, who also did not show a significant effect of teeth endodontically treated on the thickening of the maxillary sinus mucosa [29]. According to the latest research, CBCT is the best form of radiological imaging to assess the condition of endodontic treatment [43]. The compatibility of the results with the team of researchers Aksoy and Orhan results from thorough analysis of radiological examinations and proper qualification of teeth to each group.

The vast majority of authors agree that the loss of alveolar bone significantly impacts the excessive thickening of the maxillary sinus mucosa [4,11,39]. The presented study also found a significant impact of alveolar bone loss on the increase in the thickening of the maxillary sinus mucosa. The presence of severe periodontal bone loss may cause a local reaction of the sinus mucosa, such as edema, lymphocyte migration, fibrosis, or cell destruction. The effect of these changes may result in the thickening of the maxillary sinus mucosa [44]. In addition, the sinus may become infected as a result of bacterial infection existing in deep periodontal pockets. This fact is explained because the bottom of the maxillary sinus is perforated by numerous blood and lymphatic vessels, which promotes close contact between the maxillary sinus and the periodontal ligament in the bone region of adjacent teeth [13]. Another factor contributing to infection is the blood vessels supplying the periodontium and the teeth in the maxilla, which create anastomoses with the arteries responsible for vascularization of the maxillary sinus [13]. The close distance between the root apex of the teeth in the maxilla and the bottom of the maxillary sinus can cause numerous complications during the formation of odontogenic infections or dental treatment. According to the authors of Roque-Torres and Goller-Bulut et al., the closer the distance between the root apex and the bottom of the maxillary sinus, the more significant impact on maxillary sinus inflammation, such as thickening of its mucosa can be generated [4,45]. Huang et al. proved that maxillary sinus membrane thickening is significantly associated with periapical lesions and periodontal bone loss [46]. Similar conclusions were presented in this study, determining the negative correlation of the distance between the roots of the teeth and the bottom of the maxillary sinus and the thickening of the maxillary sinus mucosa.

In the present study, the root apices of teeth in pathological conditions were shown to be closer to the maxillary sinus floor than healthy teeth. In addition, both the effect of the clinical condition and the distance of the tooth root apexes were found to increase the thickening of the maxillary sinus floor. Thus, it can be assumed that the effect of the clinical condition of the tooth has a more substantial effect on the occurrence of thickening of the maxillary sinus mucosa due to its simultaneous effect on the distance of the tooth root tips from the maxillary sinus floor.

The clinical implications of this study should be emphasized. Sinus diseases, manifested mainly by inflammation of the maxillary sinus, thickening of the sinus mucosa, could be a problem when patients need sinus lifting surgery with augmentation of bone grafts or the placement of dental implants [15,16,17,18]. Therefore, a thorough radiological diagnosis, including evaluation of the status of the maxillary sinuses, should be performed before the planned implant surgery. In addition, alveolar bone loss-like periodontal disease, correlates positively with the thickening of the maxillary sinus mucosa. Therefore, periodontal treatment should be considered before planned implantation.

The limitations of this study are that it is a retrospective study, performed only on CBCT images and patient medical records. Therefore, it was not possible to extend the physical examination or the interview. In addition, it was not known precisely when dental treatment was performed, and there were no further appointments to see if the thickening of the maxillary sinus mucosa was changing.

## 5. Conclusions

The presented study revealed a significant influence of periapical lesions, inappropriate endodontic treatment, severe caries, and extracted teeth on the presence of increased thickening of maxillary sinus mucous membrane. In addition, an increase in the distance between root apices and maxillary sinus floor triggered a significant reduction of maxillary sinus mucous membrane thickening. The presence of periodontal bone loss significantly increases maxillary sinus mucous membrane thickening.

## Figures and Tables

**Table 1:** Baseline characteristics.

Sex				N		%	
Woman				50		50%	
Man				50		50%	
Wiek [lata]							
N	Mean	SD	Median	Min	Max	Q1	Q3
100	46,63	15,5	47	22	84	33	60

N—number of patients, SD—standard deviation, min—minimum value, max—maximum value, Q1—first quartile, Q3—third quartile.

**Table 2:** Comparison analysis of mucosal thickening according to odontogenic condition.

Odontogenic Condition	Mucosa Thickening (mm)			
**Group**	**Mean + SD**	**Median**	**Quartiles**	*p* < 0.001 NND P > NE,R,C,I,E,H NE > R,C,I,E,H R > C,I,E,H C > I,E,H
H (N = 468)	2.87 ± 5.04	1.5	0.7–2.5	
P (N = 104)	12.35 ± 10.12	10.05	4.45–15.48	
C (N = 133)	6.38 ± 7.07	3.7	2.2–7.5	
I (N = 41)	3.8 ± 4.99	1.7	1.1–5.4	
R (N = 366)	6.46 ± 9.07	2.1	1.3–7.82	
E (N = 49)	3.25 ± 4.72	1.6	0.9–3.2	
NE (N = 39)	6.86 ± 8.9	3.5	2.05–8.4	

Key—NND—non-normal distribution of data, N—number of teeth in each group, *p*—level of significance, Kruskal–Wallis test + results of posthoc analysis (Dunn’s test).

**Table 3:** Comparison analysis of mucosal thickening according to periodontal bone loss.

Results of Analysis of Mucosal Thickening According to Periodontal Bone Loss				
Tooth Number	Correlation Coefficient	*p*	Correlation Current	Strength of Correlation
18	0.314	*p* = 0.154 NND	---	---
17	0.41	*p* < 0.001 NND	Positive	Weak
16	0.22	*p* = 0.078 NND	---	---
15	0.194	*p* = 0.116 NND	---	---
14	0.299	*p* = 0.017 NND	Positive	Very weak
13	0.248	*p* = 0.02 NND	Positive	Very weak
23	0.115	*p* = 0.283 NND	---	---
24	0.344	*p* = 0.003 NND	Positive	Weak
25	0.17	*p* = 0.177 NND	---	---
26	0.147	*p* = 0.288 NND	---	---
27	0.239	*p* = 0.045 NND	Positive	Very weak
28	0.322	*p* = 0.083 NND	---	---

Key: NND—non-normal distribution of data, *p*—level of significance, Spearman’s rank correlation coefficient.

**Table 4:** Comparison analysis of mucosal thickening according to anatomic relation between teeth and maxillary sinus.

Results of Analysis of Mucosal Thickening According to Anatomic Relation between Teeth and Maxillary Sinus				
Tooth Number	Correlation Coefficient	*p*	Correlation Current	Strength of Correlation
18	−0.121	*p* = 0.439 NP	---	---
17	0.041	*p* = 0.726 NP	---	---
16	−0.315	*p* = 0.009 NP	Negative	Weak
15	−0.297	*p* = 0.014 NP	Negative	Very weak
14	−0.168	*p* = 0.163 NP	---	---
13	0.027	*p* = 0.801 NP	---	---
23	−0.015	*p* = 0.886 NP	---	---
24	0.026	*p* = 0.823 NP	---	---
25	−0.051	*p* = 0.674 NP	---	---
26	−0.179	*p* = 0.176 NP	---	---
27	−0.329	*p* = 0.004 NP	Negative	Weak
28	0.136	*p* = 0.345 NP	---	---

Key: NND—non-normal distribution of data, *p*—level of significance, Spearman’s rank correlation coefficient.

**Table 5:** Comparative analysis of the average mucosal thickening, classified in a three-grade scale and odontogenic condition.

Teeth Group	Mucosal Thickening Grade 1	Mucosal Thickening Grade 2	Mucosal Thickening Grade 3	*p*
H	305 (52.59%)	134 (31.02%)	29 (15.43%)	<0.001
P	6 (1.03%)	46 (10.65%)	52 (27.66%)	
C	28 (4.83%)	83 (19.21%)	22 (11.70%)	
I	23 (3.97%)	15 (3.47%)	3 (1.60%)	
R	179 (30.86%)	116 (26.85%)	71 (37.77%)	
E	29 (5.00%)	16 (3.70%)	4 (2.13%)	
NE	10 (1.72%)	22 (5.09%)	7 (3.72%)	
All teeth	580 (48%)	432 (36%)	188 (16%)	

## Data Availability

Data available on request.
